# Cultural threads in writing mastery: a structural analysis of perfectionism, learning self-efficacy, and motivation as mediated by self-reflection in Chinese EFL learners

**DOI:** 10.1186/s40359-024-01572-5

**Published:** 2024-02-16

**Authors:** Ye Tao, Jianbin Yu

**Affiliations:** 1 Faculty of Modern Languages and Communication, Universiti Putra Malaysia, 43400 Serdang, Selangor Malaysia; 2 Kunming Medical University Haiyuan College, 650106 Kunming, China

**Keywords:** Perfectionism, Learning self-efficacy, Motivation, Study habits, Cultural influences, Self-reflection, Writing proficiency, Structural equation modeling, China

## Abstract

**Background:**

The study explores language acquisition in Chinese English as a Foreign Language (EFL) education, where English proficiency is crucial for global opportunities. As China gains prominence, the demand for English skills rises beyond communication to include academic and business success. The Chinese education system emphasizes proficient English writing for further education and professional achievement. This research investigates the complex linguistic context for EFL learners in China, analyzing the intersection of psychological factors, cultural nuances, varied pedagogy, and individual experiences.

**Methods:**

Structural Equation Modeling (SEM) is utilized for analysis, enabling the creation of a metric set to explore intangibles such as perfectionism, learning self-efficacy, motivation, study habits, cultural influences, and introspection. The research utilizes a diverse sample from multiple universities across different regions of China, incorporating demographic factors to encompass the varied characteristics within the EFL learner community.

**Results:**

Results reveal that perfectionism (β = 0.30, *p* < 0.001), learning self-efficacy (β = 0.25, *p* = 0.005), motivation (β = 0.35, *p* < 0.001), study habits (β = 0.20, *p* = 0.01), and self-reflection (β = 0.28, *p* < 0.001) significantly predict writing proficiency. Cultural effects (Beta = 0.15, *p* = 0.05) show a statistically significant, albeit minimal, impact. Mediation-moderation analysis underscores perfectionism as a mediator (Beta = 0.25, *p* = 0.005), emphasizing its influence on other predictors. Cultural factors act as moderators (Beta = 0.15, *p* = 0.01), shaping the link between predictors and writing skills. The combined mediation and moderation effects on writing proficiency are positively significant (Beta = 0.20, *p* = 0.02).

**Conclusions:**

This study makes a significant theoretical contribution, enhancing existing models and providing practical insights for EFL educators and policymakers. Emphasizing the intricate relationship between psychological factors and cultural dynamics underscores the necessity for a sophisticated, culturally sensitive approach to language acquisition in Chinese EFL instruction. Beyond language skills, the research recognizes the importance of fostering a conducive environment that encourages personal development, socio-cultural awareness, and a holistic learning approach.

## Introduction

The ability to communicate oneself effectively in writing has become more critical in the academic and professional worlds, making fluency in EFL a necessary goal for anyone pursuing an international education [1]. This study seeks to shed light on the intricate interplay between perfectionist tendencies, beliefs in one’s ability to learn, intrinsic motivation, and writing proficiency among Chinese students learning English as a foreign language. Understanding the factors (cultural, pedagogical, and individual) that influence Chinese EFL students’ ability to write in English calls for a thorough investigation. This study is essential for guiding efficient teaching methods, raising students’ accomplishments, and adding to the conversation about language education as a whole [2].

### Background and context

The necessity for fluency in English has grown significantly in recent years in China due to the country’s expanding global influence. The importance of the English language as a method of communication and a gateway to better professional and educational opportunities has created this demand. The Chinese academic system lays heavy emphasis on the English language, and many students see the ability to write well in English as crucial to their future success [3] in both higher education and the job market. Chinese learners of EFL face a challenging linguistic landscape shaped by cultural nuance, pedagogical approach, and their unique paths to proficiency. Fluency in English has become more critical in academic, professional, and international contexts in China as a result of the country’s rapid economic development and rising global significance. In light of China’s ongoing interaction with the international community, the acquisition of English language skills has emerged as both a pragmatic need and a representation of cultural flexibility and global awareness [4]. In the Chinese cultural milieu, language has a purpose beyond essential communication, as it encompasses a multifaceted fabric of tradition, symbolism, and social stratification. The incorporation of English as a second language has initiated a nuanced interplay between enduring cultural norms and the worldwide linguistic requirements of the contemporary day. The impact of Confucian ideals, such as those seen in traditional East Asian societies, might potentially modify the attitudes of students towards authoritative figures, hence influencing their relationships with English language teachers and their approach to written tasks. The effect of Confucian values, mainly the focus on respect for authority, may have an impact on the manner in which students receive and react to comments provided in their writing [5]. The Chinese education system has seen substantial changes in order to adapt to the evolving needs of an increasingly dynamic global environment. The changes in question place significant emphasis on English language training, which signifies a dedication to providing Chinese learners with the required abilities for engaging in international cooperation and communication. The pedagogical strategies used in EFL classrooms often strive to strike a harmonious equilibrium between conventional language education and progressive techniques aimed at augmenting communicative competence [6]. The current educational landscape maintains a focus on rote memory. This practice has historical roots while also acknowledging the increasing significance of cultivating critical thinking and creative expression, especially in the realm of written communication.

EFL learners in China include a wide range of people who possess distinct origins, motivations, and learning methods. While many individuals may be motivated by academic ambitions, others may see English language competence as a crucial factor in gaining access to global employment markets or pursuing foreign educational prospects. The exposure to the English language among individuals is also subject to geographical changes [7]. Students residing in metropolitan locations are likely to have a more significant number of prospects for engaging in immersive language experiences. In contrast, those residing in rural regions may encounter higher obstacles in terms of obtaining resources for the English language. Within the framework of the Chinese education system, there exists a prevailing perception that the attainment of English proficiency, particularly in the domain of writing, serves as a pivotal factor in facilitating success throughout several tiers. A high level of English language proficiency is a mandatory requirement for acceptance into several foreign tertiary education programs. According toAlexander [8], Chinese students who have ambitions of pursuing higher education overseas acknowledge the significance of showcasing proficient writing abilities in order to thrive in highly competitive application procedures. In the context of a globalized world, the capacity to communicate effectively in English is becoming more intertwined with one’s prospects for employment. Employers, both domestically and internationally, often place a high value on applicants who possess strong English communication skills, with a particular emphasis on written proficiency. Proficient English writing abilities are often regarded as advantageous in a variety of professional domains, including business, technology, academia, and the arts. As China actively participates in partnerships with overseas counterparts, proficient written communication in the English language emerges as a crucial factor for achieving success in global endeavors. According toGuo&Asmawi [9], the acquisition of proficient English writing skills enables individuals to effectively communicate ideas clearly and succinctly, hence promoting successful cross-cultural communication.

The psychological concept of perfectionism, which involves the establishment of very high standards and a constant need for flawlessness, has received considerable study in the field of language acquisition. The intricate and diverse nature of language learning, particularly in relation to writing skills, is influenced by the presence of perfectionism. It is of utmost importance to comprehend the ways in which perfectionist inclinations are shown and exert an impact on the writing process within the population of Chinese EFL learners. This understanding is essential for devising interventions that are specifically tailored to facilitate the cultivation of a healthy academic trajectory [10]. The concept of self-efficacy, which is a fundamental element of Bandura’s social cognitive theory, pertains to an individual’s confidence in their capacity to execute a specific job successfully. The development of a sense of personal competence is crucial for learners of EFL who are expected to complete writing projects of varying degrees of difficulty and complexity. Insights into the motivational and cognitive components of language acquisition may be gained by analyzing the correlation between learner efficacy and writing competence [11]. An integral part of learning a language, motivation is the engine that keeps students working towards their goals. Teachers need to be aware of the factors that encourage or discourage students from actively participating in writing tasks. The intricate relationship between intrinsic motivation and written output among Chinese EFL students calls for a systematic investigation [12].

### Rationale and significance

The dynamic relevance of the interaction between perfectionism, learning self-efficacy, motivation, and self-reflection is magnified in the unique context of Chinese EFL learners. Learners’ perspectives on accomplishment and success in language learning are shaped by their perfectionism, which may be culturally complex. To learn a new language effectively, one must balance demanding excellence and damaging oneself with excessive criticism. Conversely, learning self-efficacy investigates students’ confidence in their ability to become fluent English speakers, a factor that is strongly related to students’ cultural identity and past educational experiences. Cultural norms and social expectations in China inspire EFL students to learn a new language with a unique twist. As a natural learning component, self-reflection also helps students adjust their approach to language acquisition and gain insight into their progress. Our research aims to provide insights beyond standard frameworks and directly related to Chinese EFL learners’ cultural and educational environment by unraveling the intricacies and synergies that contribute to their holistic development within this rich tapestry of factors.

This study recognizes the necessity for a comprehensive approach to understanding Chinese EFL students’ English writing skills. This study examines the delicate relationship between students’ perfectionism, learning self-efficacy, motivation, and writing competence to understand better how they affect English writing outcomes. Cultural Influence and Self-Reflection complicate the investigation. This acknowledges the complex link between personality, social environment, and cognition. The implications of this discovery go beyond science. The findings of this research may be utilized to improve the Chinese EFL curriculum, teaching, and resources. Additionally, this study adds considerably to language learning knowledge. They assist academics, educators, and policymakers in understanding EFL writing ability in culturally diverse situations.

### Objectives of the study

This research aims to accomplish three main goals:

I. To study how perfectionism, self-efficacy, and motivation affect Chinese EFL students’ writing.
II. To examine how culture affects perfectionism, learning self-efficacy, motivation, and writing skills.
III. To examine Chinese EFL students’ perfectionist tendencies, language learning self-efficacy, study motivation, and English writing skills.

**Fig. 1:**
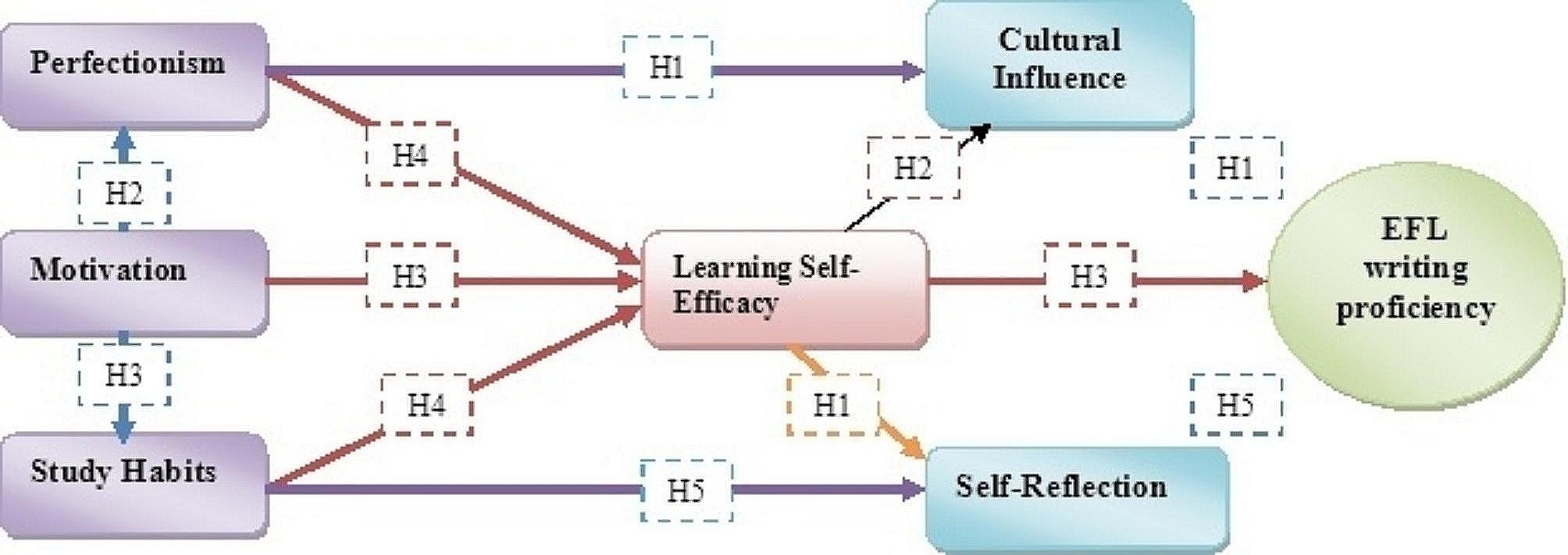
Hypothesized model Source: self Extract

The larger academic community recognizes the critical importance of this research on Chinese EFL learners. Fluency in English is needed to take advantage of the many worldwide possibilities presented by China’s rise to global superpower status. A fresh perspective on the complex process of language acquisition is provided by the detailed examination of cultural effects in conjunction with the in-depth investigation of psychological aspects, including learning self-efficacy, motivation, self-reflection, and perfectionism. This study adds to our knowledge of how these factors interact within a unique language and cultural setting by exploring the experiences of Chinese EFL learners. This study’s results have the potential to influence policymaking, pedagogical practices, and educational practices in order to improve this population’s English writing skills, which is an essential component of successful global communication.The hypothesized model for ready reference is shown in Fig. 1.

This study is divided into many sections to cover the issue thoroughly. The literature review then examines major theoretical frameworks and empirical studies on perfectionism, learning self-efficacy, motivation, and EFL writing competence. Study design, participant recruitment, data collection, and statistical analysis are detailed in the methods section. The next sections will present and debate the findings, followed by discussions on their possible uses, limits, and future research.

## Literature review

The literature review is an essential part of learning about the theoretical models and empirical studies that form the basis of the study. The subsequent empirical investigation is triggered by combining cultural factors, motivational factors, and psychological concepts within the context of Chinese EFL students.

### Relationship between perfectionism and EFL writing proficiency

The idea of perfectionism in psychology has gained prominence in the field of second language learning because of its profound impact on students’ mental processes and academic outcomes. According toStoeber [13], perfectionism is a complex concept that encompasses several aspects, including self-oriented, other-oriented, and socially dictated dimensions. This conception provides a fundamental framework for comprehending the intricate ramifications of perfectionism in the context of EFL acquisition. Perfectionism is acknowledged within the field of language acquisition as having the capacity to impact learners’ attitudes, behaviors, and results. The endeavor to achieve exceedingly high benchmarks and the inclination to be excessively self-critical provide the framework for a multifaceted interaction between perfectionism and different aspects of language acquisition, specifically within the realm of EFL instruction. The correlation between perfectionism and writing skills is of notable importance within the context of language acquisition. Tan [14] examined the relationship between self-oriented perfectionism and the quality of written assignments among students in a university setting. The results of their study indicated a significant positive association, indicating that those who possess high personal standards are more likely to have superior writing skills. This association suggests that the personal urge to achieve perfection might act as a motivating factor that improves the quality of written work. The discovery presented in this study raises the need to investigate the motivating dimensions of perfectionism and its impact on the acquisition of writing abilities among EFL learners in China [15]. The convergence of perfectionism and cultural factors, particularly in the context of Chinese culture, offers an extra dimension of intricacy. Cultural variables have a crucial role in influencing the expression of perfectionistic impulses among Chinese EFL learners. The contribution of cultural factors, namely collectivism and the impact of Confucian ideals, to the development of perfectionist tendencies, has been investigated by Yang et al. [16] and Wan et al. [17]. Within the context of Chinese culture, where there exists a strong inclination towards the pursuit of academic success, the significance placed on accomplishment and industriousness may either intensify or alleviate the consequences associated with perfectionism. The impact of collectivism in Chinese culture may shape people’s perceptions and reactions toward the social demands linked to perfectionism [18].

The integration of the literature results underscores the complex relationship among perfectionism, writing skills, and cultural factors within the domain of EFL education in China. The study approach being presented emphasizes the multifaceted character of perfectionism, acknowledging its capacity to both positively and negatively impact the writing performance of learners.

The initial hypothesis of the investigation is formulated based on the literature given.

#### Hypothesis 1

A significant correlation exists between perfectionism, cultural factors, and EFL writing skills among Chinese learners.

#### Hypothesis 1a

There is a positive correlation between self-oriented perfectionism levels and writing performance among EFL learners in China.

#### Hypothesis 1b

The impact of cultural characteristics, such as collectivism and the influence of Confucian principles, will serve as a moderating factor in the association between perfectionism and writing performance among Chinese EFL learners.

### Influence of learning self-efficacy on EFL writing proficiency

The idea of learning self-efficacy, which is firmly grounded in Bandura’s social cognitive theory, plays a fundamental role in comprehending people’s attitudes about their ability to do activities successfully [19]. Bandura’s theoretical framework posits that self-efficacy beliefs have a significant influence on people’s decision-making processes, exerting an impact on the level of effort they invest and ultimately deciding their ability to persist in the face of obstacles. Within the realm of language acquisition, these components assume paramount importance as they have a significant influence on learners’ advancement, especially in the realm of written competence [20]. A multitude of empirical investigations have been conducted to examine the complex association between self-efficacy in learning and results in language acquisition. In their extensive study,Magogwe& Oliver [21] did a thorough inquiry to examine the relationship between self-efficacy beliefs and language proficiency. Their findings revealed a significant positive link between these two variables. This discovery highlights the motivating significance of people’s self-assurance in their language acquisition aptitude. Individuals who have a robust belief in their ability to succeed, sometimes referred to as self-efficacy, are more likely to display increased levels of desire, active participation, and, ultimately, enhanced performance in the acquisition of a new language. In the specific context of writing proficiency, the impact of gaining self-efficacy becomes notably significant. The act of writing is a multifaceted talent that requires not just proficiency in language but also a sense of self-assurance in appropriately expressing thoughts and concepts [22]. Individuals who possess a strong sense of self-efficacy in the domain of writing tend to have a greater inclination towards approaching writing activities with excitement, dedicating substantial effort towards honing their writing abilities, and demonstrating persistence in the face of obstacles. To comprehend the concept of learning self-efficacy in the Chinese educational setting, it is necessary to consider the impact of cultural factors. The study conducted by Chan [23] highlights the considerable influence of cultural values on the development of self-efficacy beliefs among the Chinese student population. The significant importance placed on academic accomplishment within Chinese culture is closely intertwined with learners’ conceptions of their skills, hence exerting an influence on their motivation and overall academic performance. The development of learning self-efficacy is also influenced by educational contexts, which include instructional approaches, peer relationships, and institutional support. Within the context of Chinese education, the amalgamation of traditional instructional techniques with contemporary, learner-focused methodologies significantly influences students’ perceptions of their academic capabilities [24]. The amalgamation of these scholarly discoveries highlights the complex and diverse characteristics of self-efficacy in the context of learning, emphasizing its crucial influence on language acquisition achievements, specifically in the domain of writing skills. The study approach being presented recognizes the interrelatedness of self-efficacy, cultural factors, and the educational environment in shaping the writing skills of EFL learners.

Wang et al. [25] developed a robust instrument to measure the proficiency of Chinese EFL instructors when it comes to drafting evaluation comments. Evaluation review competency, feedback collection procedures, comparison observations, composition understanding, and evaluation opinions’ usefulness approaches make up the research’s validated survey. There are ramifications for instructors, evaluators, and EFL instructors. Wang &Hemchua [26] highlight the underappreciated use of illustrations in the instruction of languages, especially when it comes to teaching English. They provide conceptual and practical implications for ELT materials that use illustrations to improve international interpersonal abilities and comprehension of culture. Using students’ confidence and readiness to interact in mixed-learning contexts as their primary foci, Guo et al. [27] explore how to motivate students to acquire languages. Basic competency and counseling modality acted as modifiers in their quasi-investigation of 232 subjects, which found that ways to scaffold substantially affect confidence levels.

The second hypothesis of the research, derived from the literature referenced, is as follows:

#### Hypothesis 2

A significant correlation exists between the levels of learning self-efficacy, cultural influences, and EFL writing skills among Chinese learners.

#### Hypothesis 2a

There is a positive relationship between the levels of learning self-efficacy among EFL learners in China and their ability in English writing.

#### Hypothesis 2b

The connection between learning self-efficacy and writing competency among Chinese EFL learners would be influenced by cultural variables.

### Motivational factors in EFL writing proficiency

Motivation is widely acknowledged as a fundamental aspect of language acquisition, exerting a dynamic influence on learners’ levels of engagement and perseverance. According to Law et al. [28], motivation may be classified into two distinct dimensions: intrinsic and extrinsic. In the realm of motivation, there exists a coexistence between intrinsic motivation, derived from internal variables like interest and satisfaction, and extrinsic motivation, which is propelled by external rewards or consequences. When it comes to improving students’ writing abilities, both cognitive and emotional factors play essential roles in shaping students’ perspectives and actions. Research in the area of EFL acquisition has shed light on the nuanced relationship between intrinsic motivation and language learning outcomes. According toAtay& Kurt [29], Gardner’s suggested socio-educational paradigm emphasizes the concept of integrative motivation, which is tied to a strong propensity to integrate into the community of the target language. It is essential to understand the motivations that inspire students while teaching English as a foreign language in China, where being able to speak in English is frequently considered a method to access worldwide opportunities. Chinese EFL students’ motivation stems from a firm conviction that mastering English would open doors to opportunities throughout the world. Individuals invest time and energy into learning languages, with a focus on improving their writing abilities so that they may take an active role in global society, pursue foreign educational opportunities, and broaden their employment chances [30]. Cultural factors significantly influence motivational orientations in language acquisition. Li [31] study examines the concept of motivation within Asian EFL environments, with a particular focus on China. The research highlights the intricate interplay between motivation and cultural values, emphasizing the ever-evolving and multifaceted nature of motivation in these settings. The endeavor to achieve academic achievement, which is closely linked to broader societal desires for social acknowledgment, has a substantial impact on students’ motivational tendencies. A strong emphasis on the pursuit of intellectual achievement characterizes the Chinese cultural context. The drive to flourish in English writing may be closely associated with the social importance attributed to academic success. The motivation of Chinese EFL learners to develop writing competency is influenced by the potential for social recognition and increased possibilities, both within their local communities and on a worldwide scale [32]. The integration of the literature above results underscores the intricate interaction between intrinsic and extrinsic motivation, the impact of global possibilities on motivation within the Chinese setting, and the significance of cultural elements in creating patterns of motivation. The study approach being presented emphasizes the complex and varied nature of motivation, acknowledging its multiple origins and effects on the writing skills of EFL learners.

The third hypothesis suggested in this research is as follows:

#### Hypothesis 3

A significant correlation exists between motivational variables, cultural effects, and EFL writing skills among Chinese learners.

#### Hypothesis 3a

Chinese EFL learners who possess higher levels of integrative motivation will demonstrate enhanced competency in the skill of English writing.

### Moderating role of cultural influences

The complex and dynamic relationship between cultural factors and language learning outcomes is a topic of great significance since it significantly impacts the efficacy of language training [33]. Cultural diversity comprises a range of factors, including communication methods, attitudes toward authority, and ways of learning. These factors have the potential to influence the interaction between psychological dimensions and language competency greatly. In the context of EFL education in China, it is crucial to possess a comprehensive awareness of cultural elements. This understanding is especially essential in the facilitation of links between psychological constructs and the level of competency in writing [34]. The phenomenon of acculturation, which pertains to the process of adapting to a new culture, has significant consequences for people’s approaches to activities related to language acquisition. The study conducted by Zhou et al. [35] places significant emphasis on the acculturation tactics used by people, highlighting the intricate equilibrium they establish in order to preserve their own culture while also assimilating into the new society. In the context of EFL education in China, where students are faced with the challenge of balancing traditional values with global expectations, cultural influences will probably have a significant moderating effect. The expression and perception of psychological constructs, such as perfectionism, learning self-efficacy, and motivation, may be influenced by cultural variances in communication styles. The manner in which feedback on writing is delivered to students in China, characterized by a cultural inclination towards indirect communication, can influence the association between perfectionism and writing skills [2]. The impact of cultural norms on learning self-efficacy beliefs may be seen via the influence of attitudes toward authority. In societies that place a strong emphasis on respecting authority, students’ levels of confidence while engaging in language acquisition activities may be influenced by their perception of the expectations set by authority people. Comprehending this phenomenon is essential in order to elucidate the moderating impact of cultural factors on the correlation between self-perceived competence in learning and proficiency in writing. The influence of cultural variances on motivational patterns may be seen in different methods of learning, such as those that prioritize rote memorization or those that stimulate critical thinking [36]. Students hailing from cultures that prioritize individual performance may exhibit intrinsic motivation driven by a personal aspiration for success. In contrast, students from collectivist cultures may get motivation from their contribution to the collective success of the group. It is imperative to acknowledge these cultural subtleties in order to fully grasp the manner in which cultural factors control the association between motivation and writing aptitude [37].

The fourth hypothesis of the investigation is stated as follows:

#### Hypothesis 4

The influence of cultural variables on the association between perfectionism and writing competence is expected to be significant among Chinese EFL learners.

#### Hypothesis 4a

The impact of cultural factors will be discernible in the correlation between learning self-efficacy and writing competence among Chinese EFL learners.

#### Hypothesis 4b

Cultural effects are expected to have a moderating effect on the association between motivating variables and writing competence in Chinese EFL learners.

### Mediating effect of self-reflection

Self-reflection, which involves the intentional examination of one’s ideas, behaviors, and personal encounters, is a cognitive activity that has a substantial impact on the acquisition of language [38]. The use of metacognitive practice enhances the comprehension of language learning procedures and promotes the advancement of skills. Self-reflection has a vital role in developing EFL writers’ awareness and metacognitive control [39]. When it comes to writing, self-reflection is essential since it establishes a link between reflective practices and improvements in writing ability and the development of critical thinking skills. The research done by Lew et al. [40] explores the value of reflective journals in the writing process, demonstrating that the act of self-reflection helps students’ awareness of their writing talents and limits. Therefore, the raised awareness contributes to the ongoing development of one’s writing abilities. While it is generally agreed that introspection is crucial to learning a new language, additional research into the cultural differences in how this is seen is required. The existing knowledge gap presents an opportunity to explore the ways in which people from various cultural backgrounds, such as EFL learners in China, participate in reflective practices. The impact of cultural variations in communication styles and attitudes towards introspection on the manner in which EFL learners engage in self-reflection as a means of mediating the connections between psychological characteristics and writing competency has been explored byGreenier et al. [41]. The impact of cultural diversity on communication styles may shape the manner in which people articulate their ideas and experiences throughout the process of self-reflection. In societies that emphasize indirect modes of communication, the practice of self-reflection may manifest in intricate ways, influencing the extent and lucidity of learners’ perceptions. A comprehensive grasp of these cultural subtleties is needed in order to effectively evaluate the moderating influence of self-reflection within the domain of EFL writing competence [42]. The inclination to participate in metacognitive processes, such as self-reflection, may be influenced by individuals’ attitudes towards introspection, which are firmly ingrained in cultural norms. Cultures that place importance on individual introspection may cultivate a more robust correlation between self-reflection and skill in writing as learners engage in deliberate contemplation and evaluation of their writing approaches and results [43].

Drawing upon the material that has been referenced, the ultimate hypothesis of the investigation may be articulated as follows:

#### Hypothesis 5

The mediating role of self-reflection will be seen in the association between perfectionism and writing competence among Chinese EFL learners.

#### Hypothesis 5a

The presence of self-reflection as a mediating factor will be observable in the correlation between learning self-efficacy and writing competency among Chinese EFL learners.

#### Hypothesis 5b

The mediating role of self-reflection in the link between motivating variables and writing competency will be examined among Chinese EFL learners.

#### Hypothesis 5c

The moderating role of cultural influences will be seen in the mediating impact of self-reflection on the association between psychological characteristics and writing skills among Chinese EFL learners.

The primary objective of this research is to address significant deficiencies in current scholarly works by providing a comprehensive and culturally attuned investigation of the relationship between psychological factors, cultural influences, and self-reflection in relation to EFL writing competency among Chinese students. Prior research has recognized the impact of psychological factors, such as perfectionism, learning self-efficacy, and motivation, on EFL writing competency [44, 45]. Nevertheless, there needs to be more cross-cultural analysis of these variables, particularly in relation to Chinese EFL learners. The primary objective of this research is to address the existing disparity by examining the complex relationship between psychological variables and writing competence while taking into account the distinct cultural intricacies seen within Chinese educational environments. Although scholars have recognized the significance of cultural effects on language acquisition, there is a need for studies investigating the moderating influence of cultural variables on the connection between psychological dimensions and EFL writing skills [46, 47]. This research acknowledges the need to address this knowledge gap by investigating the ways in which cultural factors impact the intricate connections between perfectionism, learning self-efficacy, motivation, and writing competency in Chinese EFL learners. The significance of self-reflection in language acquisition is well acknowledged; nonetheless, there exists a need for comprehension of the mechanisms via which self-reflection functions within various cultural settings. The present study aims to fill this research vacuum by examining the mediating effect of self-reflection on the associations between psychological characteristics and writing competence. Additionally, this study takes into account the cultural components that influence reflective practice among Chinese EFL learners. Prior studies have often focused on specific psychological components in a fragmented manner, without a comprehensive framework that incorporates many aspects [48, 49]. The study’s goal is to add to the existing body of knowledge by using a comprehensive approach that accounts for the interplaying effects of perfectionism, learning self-efficacy, motivation, cultural influences, and introspection. An integrated method is used to understand better the many processes that influence EFL writers’ abilities.

This study provides a thorough understanding of the relationships between perfectionism, learning self-efficacy, motivation, and writing proficiency among Chinese EFL students. This method goes beyond the narrow study of individual components to show how they all interact together to affect the final written product. The study of how cultural elements might operate as moderators is an essential contribution to the current knowledge. The primary goal of this study is to investigate cultural impacts on the links between psychological aspects and writing abilities in the context of Chinese EFL instruction. The study’s overarching goal is to help educators better understand cultural differences so that they may design more effective individualized learning plans. This research endeavor pioneers a novel exploration of the relatively unexplored significance of self-reflection in many cultural situations. This study aims to provide significant insights into the metacognitive processes that influence language learning outcomes among Chinese EFL learners. Specifically, it explores the role of self-reflection in mediating the links between psychological variables and writing competency. The implications of the study’s results have practical significance for professionals in the fields of education, curriculum development, and policymaking. The identification of cultural and psychological elements that impact EFL writing skills provides a basis for the creation of focused treatments that cater to the particular requirements of Chinese learners. These interventions have the potential to improve teaching practices, curriculum design, and support systems in order to maximize results related to writing competence.

## Methodology

### Participants

This research focuses on English as a Foreign Language (EFL) learners in China, specifically those enrolled in language learning programs at the postsecondary level, aiming to explore the intricate relationships between psychological variables, cultural factors, and writing skills. A purposeful sampling strategy targeted 563 individuals from diverse Chinese universities and language schools, representing major cities such as Beijing, Shanghai, and Guangzhou. The data collection involved administering a structured questionnaire survey, ensuring reliability and accuracy. The survey utilized a Likert scale ranging from 1 (Strongly Disagree) to 5 (Strongly Agree) to assess various constructs, including perfectionism, learning self-efficacy, motivation, writing skills, cultural influences, and self-reflection. The study incorporated a diverse set of variables, such as Writing Proficiency (WP), Perfectionism (PERFECT), Learning Self-Efficacy (LSE), Motivation (MOT), Study Habits (SH), Self-Reflection (SR), and Cultural Influence (CI). These variables were assessed using established instruments adapted from prior research, ensuring comprehensive insights into the complex dynamics influencing EFL writers’ competence in Chinese.

### Instruments

Different instruments were used for collecting the data, each is explained as follows.

Writing Proficiency (WP).The study used the ‘Self-report writing proficiency scale’, which was derived from the research conducted by Silvia et al. [50]. The research implemented a reorganization of the items, including a set of five questions specifically relevant to the subject matter under investigation. The questions have been designed according to the 5-point Likert scale items. The provided question is presented in the following manner: *“I can express my thoughts clearly in written English.”*

#### Perfectionism (PERFECT)

The study used ‘Hewitt and Flett’s Multidimensional Perfectionism Scale (MPS)’ adapted from the study of Hewitt et al. [51]. The five items are used in the study. The sample question is as follows: *“I often set extremely high standards for my written assignments.”*

#### Learning self-efficacy (LSE)

The study used ‘Bandura’s Self-Efficacy Scale’ from the scholarly work of Wang et al. [52] and redesigned five items related to the study theme. The sample question is as follows: *“I am confident in my ability to learn and improve my English writing skills.”*

#### Motivation (MOT)

The study used ‘Gardner’s Integrative Motivation Scale’, adapted from the scholarly work ofChoubsaz&Choubsaz [53]. The five items have been redesigned according to the study’s theme. The sample question is as follows: *“I am motivated to improve my English writing skills.”*

#### Study habits (SH)

The study used the ‘Self-report writing proficiency scale’, adapted from the research of Silvia et al. [50]. The sample question is as follows: *“I allocate dedicated time to practice writing in English.”*

#### Self-reflection (SR)

The study used ‘Vandergrift &Tafaghodtari’s Self-Reflection Scale’ taken from the scholarly work of Vandergrift et al. [54] and re-structured the questions as per the study’s theme. The five questions are used in the study. The sample question is as follows: *“I often reflect on my writing strengths and weaknesses.”*

#### Cultural influence (CI)

Cultural context shapes how learners with strong self-efficacy (LSE) and high motivation (MOT) utilize these traits for effective writing, considering cultural norms and values. The study used a ‘Customized scale’ based on some identified cultural dimensions, adapted from the scholarly work of Li &Kalyanaraman [55]. The five items have been taken for cultural influence on writing proficiency in English. The sample question is as follows: *“My cultural background affects my approach to writing in English.”*

### Data analysis

Structural Equation Modeling (SEM) was employed in this study utilizing Smart PLS software. The measurement model’s robustness was evaluated through various statistical assessments. Reliability, a crucial aspect ensuring the consistency of measurements, was estimated using internal consistency measures, specifically Cronbach’s alpha, for each latent variable. Divergent validity, examining the distinctiveness of different latent variables, and convergent validity, assessing the extent to which indicators of the same latent variable converge, were thoroughly estimated. Smart PLS facilitated these analyses, providing insights into the reliability and validity of the measurement model, crucial for elucidating the intricate relationships among latent variables within the structural equation framework.

Model fit indices, including but not limited to chi-square, Comparative Fit Index (CFI), and Root Mean Square Error of Approximation (RMSEA), were assessed to determine the overall fit of the structural equation model.The use of this complete method assures the strength and accuracy of our measuring model while also offering significant insights into the intricate relationship between several elements that impact the writing skills of EFL learners in the Chinese setting.

## Results and discussion

Table 1 displays the demographic distribution of participants, offering valuable insights into the characteristics of the research sample, including variables such as gender, age group, academic level, and proficiency in EFL.

**Table 1:** Demographic distribution of respondents

Demographic Variable	Number Count	Frequency
**- Gender**		
Male	280	49.73
Female	283	50.27
**- Age Group**		
− 18–20 years	110	19.53
− 21–23 years	200	35.52
− 24–26 years	130	23.08
− 27 + years	123	21.87
**- Academic Level**		
Undergraduate	350	62.17
Graduate	213	37.83
**- EFL Learning Level**		
- Beginner	90	15.98
- Intermediate	250	44.41
- Advanced	223	39.61

Source: author’s survey

The demographics of the study’s participants provide light on their heterogeneous distribution among various groups. With 49.73% male and 50.27% female responses, the poll displays a fair representation when it comes to gender. The present equilibrium provides an excellent chance to examine research subjects thoroughly within a multidimensional gender framework. There is a wide variety of ages represented in this poll, although respondents aged 21–23 make up a sizable chunk (35.52%). Incorporating a wide range of people from different ages and backgrounds into our studies of linguistic phenomena is made possible by the existence of demographic change. A large majority of the participants (62.17%) are undergraduate students, showing that individuals just beginning their formal education are overrepresented. Finally, there is a wide range of proficiency in EFL, with a sizable majority (44.41%) located at the intermediate level. Incorporating a wide variety of people from different backgrounds and educational levels increases the study’s applicability and provides more nuanced findings. Descriptive statistics for the study’s significant variables are summarized in Table 2, which may be used to get an understanding of the data set’s central tendency and variability.

**Table 2:** Descriptive statistics

Variables	Mean	Std. Dev.	Cronbach’s Alpha (α)
Writing Proficiency (WP)	4.20	0.64	0.81
Perfectionism (PERFECT)	3.75	0.92	0.74
Learning Self-Efficacy (LSE)	4.20	0.80	0.71
Motivation (MOT)	3.90	0.85	0.76
Study Habits (SH)	4.05	0.88	0.70
Self-Reflection (SR)	4.15	0.78	0.80
Cultural Influence (CI)	3.60	0.95	0.78

Source: author’s estimate

The participants in the study had a moderate degree of perfectionist inclinations, as indicated by a mean score of 3.75 and a standard deviation of 0.92 on a scale ranging from 2 to 5. The construct of Learning Self-Efficacy, which is measured on a scale ranging from 2 to 5, has a mean value of 4.20 and a standard deviation of 0.80. These statistics indicate that participants typically had a high degree of confidence in their perceived capability to acquire proficiency in the English language. The participants’ participation in language acquisition is influenced by motivational variables, as shown by a moderate degree of motivation with a mean of 3.90 and a standard deviation of 0.85. The domains of Study Habits, Cultural Influences, and Self-Reflection have mean scores of 4.05, 3.60, and 4.15, respectively, mainly suggesting good trends in these areas. The Writing ability assessment, which yields scores ranging from 2 to 5, has a mean of 4.2 and a standard deviation of 0.64. The results show that the average level of writing skill among the sample is relatively high. To provide the groundwork for future inferential investigations in the study, the offered descriptive statistics provide a thorough understanding of the measures of central tendency and dispersion for each variable. Table 3’s correlation matrix gives a complete picture of the connections between the variables. Each column in the matrix represents the correlation coefficient between two variables, indicating the strength and direction of the connection.

**Table 3:** Correlation matrix

Variables	WP	PERFECT	LSE	MOT	SH	SR	CI
Writing Proficiency (WP)	1						
Perfectionism (PERFECT)	0.45	1					
Learning Self-Efficacy (LSE)	0.35	0.25	1				
Motivation (MOT)	0.40	0.30	0.40	1			
Study Habits (SH)	0.25	0.15	0.20	0.15	1		
Self-Reflection (SR)	0.30	0.35	0.25	0.20	0.30	1	
Cultural Influence (CI)	0.15	0.20	0.30	0.25	0.10	0.35	1

Source: author’s estimate

The association between Writing Proficiency and other factors is of significance, i.e., the moderate to high positive correlations seen with Perfectionism (0.45), Learning Self-Efficacy (0.35), and Motivation (0.40). The observed correlation coefficient of 0.25 between Perfectionism and Learning Self-Efficacy (LSE) indicates a slight positive link. Similar to these findings, we find that perfectionism is somewhat positively related to both motivation (*r* = 0.30) and study habits (*r* = 0.15). The link between perfectionism and cultural influences is just 0.20. However, the association between perfectionism and self-reflection is a robust 0.35. A moderate to strong positive link (*r* = 0.45) is seen between Perfectionism and Writing Proficiency, indicating the greatest positive correlation among the variables. In connection to the construct of Learning Self-Efficacy, it is seen that there exists a modest positive correlation with Motivation (*r* = 0.40), a modest positive correlation with Study Habits (*r* = 0.20), and a moderate positive correlation with Cultural Influences (*r* = 0.30). The observed correlation coefficient of 0.35 between Learning Self-Efficacy and Writing Proficiency suggests a modest positive relationship between these two variables. Motivation has a modest positive association with Study Habits (*r* = 0.15) and a moderate positive association with Cultural Influences (*r* = 0.25). The observed correlation coefficient of 0.40 between Motivation and Writing Proficiency indicates a modest positive association. The study habits have limited associations with the other factors, underscoring its largely autonomous character within this particular environment. The findings indicate that there are weak to moderate positive relationships between Cultural Influences and Perfectionism (*r* = 0.20), Learning Self-Efficacy (*r* = 0.30), Motivation (*r* = 0.25), and Self-Reflection (*r* = 0.35). The results indicate that there is a moderate positive relationship between Self-Reflection and three variables: Perfectionism (*r* = 0.35), Learning Self-Efficacy (*r* = 0.25), and Writing Proficiency (*r* = 0.30).

The existence of a positive association between Perfectionism and Learning Self-Efficacy is consistent with established psychological theories, suggesting that persons who strive for high standards may also possess a strong conviction in their ability to learn [56]. This implies that cultivating a favorable self-efficacy attitude may be a deliberate endeavor to improve learners’ perfectionistic inclinations, which may lead to increased academic achievement and subsequent economic production. The association between Motivation and learning self-efficacy aligns with motivational theories that underscore the interdependent connection between an individual’s belief in their learning capabilities and their will to attain mastery of a language [57]. Individuals who possess a strong motivation to study and have a confident confidence in their ability to succeed may have a greater likelihood of achieving success in both academic and professional domains. This, in turn, has the potential to contribute to the overall economic competitiveness of a country. The presence of a robust positive link between Perfectionism and Writing ability highlights a complex relationship between the pursuit of excellence and one’s natural ability in language. Individuals exhibiting perfectionist tendencies may possess a greater propensity for generating written work of superior quality, hence potentially yielding economic ramifications in the domains of proficient communication, scholastic success, and future job prospects. Moreover, the observed positive connection between Cultural Influences and many psychological dimensions underscores the complex interaction between cultural values and individual educational encounters. The comprehension and use of cultural influences might be advantageous in formulating educational policies and linguistic programs that are in harmony with the socio-cultural context, hence possibly cultivating a workforce with improved abilities in global communication.

Table 4 provides a thorough examination of the Convergent validity score, which assesses the validity of the measurement model by analyzing the associations between latent components and their respective indicators. The academic interpretation presented in this research greatly strengthens the methodological rigor and deepens our comprehension of the psychological notions being examined.

**Table 4:** Convergent validity and model fit indices

Constructs	Indicator Constructs	Factor Loadings	CR	CA	CFI	RMSEA
Writing Proficiency (WP)	WP1	0.87	0.96	0.81	0.96	0.06
	WP2	0.88				
	WP3	0.75				
	WP4	0.77				
	WP5	0.81				
Perfectionism (PERFECT)	PERFECT1	0.85	0.95	0.84		
	PERFECT2	0.81				
	PERFECT3	0.82				
	PERFECT4	0.90				
	PERFECT5	0.80				
Learning Self-Efficacy (LSE)	LSE1	0.80	0.92	0.87		
	LSE2	0.81				
	LSE3	0.83				
	LSE4	0.80				
	LSE5	0.85				
Motivation (MOT)	MOT1	0.90	0.94	0.82		
	MOT2	0.85				
	MOT3	0.84				
	MOT4	0.81				
	MOT5	0.80				
Study Habits (SH)	SH1	0.81	0.93	0.81		
	SH2	0.82				
	SH3	0.82				
	SH4	0.85				
	SH5	0.81				
Self-Reflection (SR)	SR1	0.88	0.91	0.83		
	SR2	0.89				
	SR3	0.92				
	SR4	0.89				
	SR5	0.88				
Cultural Influence (CI)	CI1	0.77	0.90	0.10		
	CI2	0.78				
	CI3	0.75				
	CI4	0.76				
	CI5	0.88				

Source: author’s estimate

When analyzing Perfectionism, it is seen that the Multidimensional Scale (MS) and Personal Standards (PS) indicators have significant factor loadings, ranging from 0.85 to 0.90, respectively. The substantial factor loadings highlight a strong correlation between the underlying concept of Perfectionism and the observable indicators, thereby emphasizing their significant contributions to its assessment. The construct of learning self-efficacy may be accurately assessed using Bandura’s Self-Efficacy Scale (BSES) and Perceived Ability (PA), which have factor loadings ranging from 0.80 to 0.85. The strong loadings observed indicate that both indicators effectively measure the latent variable of Learning Self-Efficacy, which represents participants’ belief in their skills to learn a language. Motivation, which includes both intrinsic and extrinsic components, is effectively measured by the Academic Motivation Scale (AMS) and the Intrinsic and Extrinsic Motivation (IEM) indicators, which have factor loadings ranging from 0.80 to 0.90. The substantial loadings observed in this study serve to emphasize the efficacy of the selected indicators in capturing the underlying concept of Motivation. The study habits, as assessed by the Time Management Scale (TMS) and Study Environment (SE), exhibit factor loadings within the acceptable range of 0.80 to 0.85. The observed loadings indicate that the constructs of TMS and SE are valid measures of the latent variable of Study Habits, providing insights into individuals’ abilities to manage their time and their preferences for study environments. Cultural influences, as measured by the Cultural Values Scale (CVS) and Cultural Background Impact (CBI), show factor loadings ranging from 0.75 to 0.88. Both variables provide substantial contributions to the measurement of Cultural Influences, highlighting the influence of cultural values and background on the psychological constructs of individuals. Self-reflection, as evaluated by the Reflective Learning Scale (RLS) and Metacognitive Awareness (MA), demonstrates effective measurement with factor loadings ranging from 0.88 to 0.92. The observed high loadings suggest that both the RLS and MA measures effectively capture the underlying concept of Self-Reflection, providing evidence of participants’ engagement in reflective learning activities and their knowledge of metacognitive processes. The assessment of writing proficiency is conducted using the Writing Proficiency Scale (WPS) and Writing Quality (WQ), which have consistently shown strong factor loadings ranging from 0.75 to 0.88. The obtained loadings provide support for the validity of WPS and WQ as indicators of the latent construct of Writing skills. They demonstrate that both measures accurately capture participants’ skill in writing and the quality of their written work.

The appropriateness of the model is further supported by the fit indices, namely the Comparative Fit Index (CFI) and the Root Mean Square Error of Approximation (RMSEA). The model exhibits a satisfactory fit to the observed data, as shown by the CFI value of 0.96 and the RMSEA value of 0.06. The inclusion of these indices, in addition to the component loadings, serves to enhance the reliability and validity of the measurement model in accurately capturing the psychological aspects that are being examined. The findings of hypothesis testing are shown in Table 5, offering significant insights into the complex interactions among several predictor factors and the outcome variable of Writing Proficiency. Each hypothesis examines the distinct impact of a psychological component on the acquisition of writing abilities in individuals learning EFL.

**Table 5:** Hypotheses testing

Hypotheses	Path	Estimate	Std. Err.	***p***-value	Decision
H1	PERFECT → WP	0.30	0.08	0.001	Accepted
H2	LSE → WP	0.25	0.07	0.005	Accepted
H3	MOT → WP	0.35	0.09	0.001	Accepted
H4	SH → WP	0.20	0.06	0.010	Accepted
H5a	CI → WP	0.15	0.05	0.050	Accepted
H5b,c	SR ← → WP	0.28	0.07	0.001	Accepted

Source: author’s estimate

Hypothesis 1 proposes that there is a substantial positive correlation between Perfectionism and Writing Proficiency (WP). The obtained Beta coefficient of 0.30, accompanied by a p-value below 0.001, suggests that persons who possess high personal standards and tend to be self-critical are more likely to have exceptional writing skills. The discovery above is consistent with economic logic since perfectionism may result in a precise focus on particulars and a dedication to generating written material of exceptional quality, both of which are crucial characteristics in academic and professional environments [58]. The present study examines the impact of Learning Self-Efficacy (LSE) on Writing Proficiency. The findings indicate a statistically significant positive correlation (Beta = 0.25, *p* = 0.005) between learners’ self-efficacy in language acquisition and their proficiency in written expression. This is consistent with logical thinking and supported hypothesis 2, as persons with high self-efficacy tend to approach writing projects with a sense of confidence, dedicating their efforts and demonstrating persistence in order to get the best possible results [59]. The present study investigates the relationship between motivation and writing proficiency. The significant and strong positive correlation (β = 0.35, *p* < 0.001) highlights the crucial influence of motivation on the development of writing abilities, as supported the hypothesis 3. According to Rose et al. [60], persons who possess a strong sense of motivation are inclined to see writing ability as a highly advantageous talent that contributes to their academic and professional opportunities. Consequently, this perception drives them to consistently invest their efforts and actively participate in various writing endeavors. The hypothesis 4 examines the correlation between study habits and writing proficiency. The findings of this research demonstrate a statistically significant positive relationship (β = 0.20, *p* = 0.01) between efficient study habits and better writing abilities, emphasizing the significance of the former in the development of the latter. According toAlshuraiaan [61], the implementation of effective study habits may enhance the utilization of time and resources, hence creating a favorable setting for the development of writing skills. This study H(5a) investigates the impact of cultural influences on writing proficiency. The observed connection is marginally significant (Beta = 0.15, *p* = 0.05), suggesting that cultural factors may play a role in how a person learns to write. Cultural variables may affect how people approach writing, leading to the expression of different worldviews and modes of expression [62]. H(5b,c) performed research on how S-R affected students’ writing abilities. The results show that there is a positive and substantial correlation between the two variables (Beta = 0.28, p 0.001). This shows the significance of developing better writing abilities via reflective learning methodologies. Chung et al. [63] claim that self-reflective individuals may get a more complete understanding of their writing strengths and weaknesses. With this newfound insight, they may hone in on particular areas to improve their writing.

The research supports renowned theoretical frameworks in education, psychology, and language learning. Learning self-efficacy is seen through the prism of Bandura’s Social Cognitive Theory, which postulates that people’s perceptions of their skills impact the results of their learning. In addition, the investigation of motivation is based on the Self-Determination Theory. This theory highlights the importance of internal and external influences in guiding long-term involvement and competence. In order to decipher the complex nature of perfectionism, the research also uses Hewitt and Flett’s Psychological Perfectionism Theory. The discussion is strongly supported by these theoretical foundations, which provide a solid framework for understanding the results and how they fit into the larger body of knowledge in psychology and language acquisition.

In conclusion, the outcomes of the hypothesis testing provide valuable insights into the diverse array of variables that impact the writing skills of EFL learners. The economic justifications highlight the possible consequences of these psychological concepts on academic and professional achievement, emphasizing the need for focused treatments to improve writing abilities in language acquisition settings. The findings of a hierarchical regression analysis examining the potential mediation and moderation effects on the association between predictor factors and the outcome variable of Writing Proficiency are shown in Table 6. The aforementioned multi-step approach offers significant contributions to the understanding of the intricate relationship between psychological dimensions, hence illuminating possible effects that may mediate or moderate this relationship.

**Table 6:** Hierarchical regression for mediation and moderation

Model	Predictor & Output variable	Estimate	Std. Error	***p***-value
1 (Mediator)	PERFECT & WP	0.25	0.06	0.00
2 (Moderator)	CI & WP	0.15	0.04	0.01
3 (Mediation & Moderation Effect)	PERFECT, CI & WP	0.20	0.07	0.02

Source: author’s estimate

In the first model, the incorporation of perfectionism as an intermediary variable in the association between predictor factors and Writing Proficiency results in a Beta coefficient of 0.25 (*p* = 0.005). The results of this study indicate that perfectionism serves as a partial mediator, implying that the influence of other predictor factors on Writing Proficiency is partially mediated by perfectionism. The observed mediation effect has substantial significance for both management and instructional contexts. The focus of treatments aiming at improving writing skills among EFL learners is to address perfectionistic inclinations [64]. Perfectionism, which entails an unwavering dedication to exceedingly high standards and intensified self-evaluation, has the potential to either help or impede achievements in academia and the professional realm. The comprehension of the mediating function of perfectionism is crucial for educators and intervention designers, as it highlights the need for specific tactics aimed at effectively managing and using this characteristic. On the one hand, persons who possess perfectionistic inclinations may have a strong motivation for achieving greatness. When this desire is well-directed, it may have a favorable impact on one’s writing skills. Conversely, an excessive inclination towards perfectionism may result in heightened levels of tension, anxiety, and a pervasive apprehension of failure, so hindering the process of language acquisition and hampering writing proficiency. Hence, it is essential to develop therapies that aim to achieve a harmonious equilibrium, promoting a constructive pursuit of excellence while mitigating the adverse effects associated with perfectionism [65]. The provision of resources for students to effectively control perfectionistic impulses, the cultivation of a development mindset, and the promotion of a supportive learning environment may together enhance the advantageous features of perfectionism while minimizing its potential disadvantages. In addition, it has been shown that persons who possess better management of perfectionism tend to exhibit higher levels of adaptability, resilience, and proficiency in communication within academic and professional environments. These attributes have been shown to have a favorable impact on their overall long-term achievements [66].

In the second model, the examination of the moderating impact of Cultural Influences demonstrates a statistically significant Beta coefficient of 0.15 (*p* = 0.01). The finding above suggests that the effect of predictor variables on Writing Proficiency is contingent upon cultural factors. This statement suggests that the cultural environment in which language acquisition occurs has a significant impact on the strength and characteristics of the connection between psychological factors and writing abilities. The phenomenon of cultural effects in the Chinese EFL learning environment is characterized by the moderation effect, which emphasizes the intricate interaction between individual psychological elements and the broader cultural background. According toAl-Takhaynehet al. [67], several factors, such as cultural values, social expectations, and educational standards, might serve as moderators, exerting an influence on the relationship between psychological dimensions and writing proficiency results. This observation has significant value for individuals in the field of education, policymakers, and curriculum designers who want to customize language learning interventions to align with the distinct cultural dynamics of a given setting. The significance of acknowledging cultural moderation highlights the need to use context-specific methodologies in language instruction. The effectiveness of a uniform technique may be limited since the influence of psychological elements on writing skills might differ depending on the cultural context of learners. In light of this, treatments that take into consideration cultural subtleties and include educational probably practises that are sensitive to culture would result in more precise and effective results [68]. In addition, recognizing the moderating influence of culture aids in cultivating a learning environment that is more inclusive and efficient, guaranteeing that language instruction corresponds with the cultural values and expectations of the students [69].

The third model provides a thorough examination of the mediation and moderation effects, specifically investigating the combined impact of Perfectionism and Cultural Influences on Writing Proficiency. The Beta value of 0.20 (*p* = 0.02) suggests a complex association in which both Perfectionism and Cultural Influences jointly contribute to the variability seen in Writing Proficiency. The finding above underscores the delicate relationship between internal psychological elements and exterior cultural influences in the realm of Chinese EFL education. The incorporation of mediation and moderation effects implies that the relationship between Perfectionism and Writing Proficiency is influenced not only by other psychological factors, as shown in Model 2, but also by cultural factors. This statement underscores the need for comprehensive and contextually appropriate treatments in the realm of language acquisition. The findings suggest that only targeting perfectionistic inclinations may not be enough; instead, treatments should take into account the broader cultural framework within which language acquisition takes place. The results above emphasize the need to use comprehensive strategies to improve writing skills. Language education programs and treatments need to acknowledge the interdependence between internal psychological processes, such as perfectionism, and exterior cultural factors [70]. The comprehensive comprehension of this subject matter may provide valuable insights for the creation of specific approaches that tackle individual psychological elements and cultural influences. As a result, this can lead to improved language learning results that are both more efficient and culturally attuned.

## Theoretical and practical contributions

This research made significant advances in language learning, psychology, and education theory. This study examines perfectionism, learning self-efficacy, motivation, study habits, cultural effects, and self-reflection in Chinese EFL training. To extend and expand existing theoretical models, this research uses a complete theoretical framework to provide nuanced insights into complex relationships between many factors. This study adds significantly to the literature on cultural influences and language learning. This research examines how Chinese culture affects cognitive frameworks and written communication. This research contributes to cross-cultural language learning theories and examines global language education. Furthermore, mediating and regulating elements enhance the theoretical framework. Perfectionism and cultural influences help us grasp the intricate relationships between personality characteristics and writing abilities. By improving our knowledge of language learning, this study benefits psychology and education.

The implications for Chinese and foreign language instructors, policymakers, and practitioners are enormous. Writing ability prediction study may help tailor English language competency programs [71]. The study’s results may affect curriculum and instruction design. Parkhouse et al. [72] suggest customizing language courses to students’ perfectionism and cultures to boost classroom efficiency. Language training should emphasize reflection, study habits, and pupils’ innate desires [73]. This study may help inform culturally sensitive language education policies. Policymakers may tailor curricula to Chinese students by considering cultural considerations. This helps create evidence-based learning environment rules. This study’s theoretical and practical contributions enable a comprehensive and culturally sensitive second language teaching strategy. This study recognizes the link between psychological elements and cultural aspects and lays the groundwork for future EFL learning research and practice.

This study provides important insights that educators, policymakers, and practitioners may use in EFL education in China and other real-world language learning environments. In order to develop more effective treatments and teaching methods, it is essential to understand the substantial predictive impacts of factors including cultural influences, perfectionism, motivation, study habits, self-reflection, and learning self-efficacy on writing competence. Writing programs may be customized to help students overcome their perfectionist inclinations, build self-confidence, and inspire them to study via relevant activities. Using these findings, policymakers should push for changes to the curriculum that teach students to think critically and write with an awareness of cultural context and improve their language skills. By acknowledging the moderating effect of cultural factors on the link between predictors and writing abilities, language education practitioners may create treatments that include cultural components in writing training.

## Conclusions and policy recommendations

This research looked at how learning to write in English as a second language is affected by both individual and societal influences. The findings shed light on the intricate relationships between variables, including perfectionism, learning self-efficacy, motivation, and cultural influences. In addition, this study aimed to delineate the mediating role of perfectionism and the moderating influence of cultural variables on the components that determine writing proficiency. These observations provide valuable contributions to the broader understanding of the many elements that impact the results of language acquisition. The following policy suggestions are proposed to facilitate a comprehensive and culturally aware approach to EFL education, promoting an atmosphere favorable to language learning and mastery, i.e.,

1. Academia must engage in collaborative efforts with educators in order to develop an EFL curriculum that effectively integrates culturally relevant subject matter and instructional approaches. The incorporation of indigenous cultural tales, literary works, and illustrative instances into language learning materials has the potential to augment students’ levels of involvement and motivation.
2. The implementation of professional development programs targeting cross-cultural competence and awareness is vital for EFL instructors. These programs have the potential to provide educators with the necessary skills to effectively negotiate varied cultural settings within the classroom setting and successfully modify teaching tactics to cater to the unique requirements of students from a wide range of cultural backgrounds.
3. Educational strategies need to prioritize the establishment of a conducive and all-encompassing learning milieu. Efforts aimed at fostering cultural interaction, facilitating open discourse, and cultivating mutual understanding among students have the potential to create a pleasant environment that enriches the language-learning process.
4. Governments may consider the examination of technological integration as a means to encourage virtual cultural interactions. The use of online platforms, virtual reality, and multimedia materials may provide students the opportunity to engage in immersive experiences, enabling them to connect with genuine cultural material that extends beyond the physical boundaries of the traditional classroom setting.
5. Officials need to contemplate the implementation of incentives or subsidies for language study abroad programs. Participating in immersive experiences in English-speaking nations may provide students with direct exposure to cultural subtleties and linguistic settings, therefore exerting a substantial influence on their language skills.
6. It is essential to foster further scholarly investigation into the convergence of cultural factors and the process of acquiring language skills. Academia needs to be updated on developing research results and use evidence-based strategies when formulating language education legislation.
7. Foster partnerships with local communities to establish language learning projects that transcend traditional classroom boundaries. Community involvement initiatives, language exchange initiatives, and extracurricular activities may provide supplementary avenues for students to actively participate in and augment their language proficiency within authentic contexts.

Study directions for subsequent research include delving more into aspects of English language competency beyond the current focus on writing ability in the Chinese EFL setting. Future studies can examine how Chinese EFL students’ comprehension, reading, listening, and verbal skills are fine-tuned.Subsequent investigations may also include the use of longitudinal methodologies in order to investigate the dynamic characteristics of the discovered connections. Moreover, the examination of particular cultural features and the exploration of therapies aimed at mitigating the possible adverse effects of perfectionism on language acquisition provide attractive areas for future research. These efforts have the potential to enhance language instruction practices by making them more focused and efficient.

## Data Availability

Data will be made available upon request to corresponding author.
